# Frontostriatal Circuits and the Development of Bulimia Nervosa

**DOI:** 10.3389/fnbeh.2014.00395

**Published:** 2014-11-17

**Authors:** Laura A. Berner, Rachel Marsh

**Affiliations:** ^1^Department of Psychology, Drexel University, Philadelphia, PA, USA; ^2^MRI Unit, Department of Psychiatry, Division of Child and Adolescent Psychiatry, New York State Psychiatric Institute, College of Physicians and Surgeons, Columbia University, New York, NY, USA

**Keywords:** bulimia nervosa, eating disorder development, frontostriatal circuits, binge eating, purging

## Abstract

Bulimia nervosa (BN) is characterized by both recurrent episodes of binge eating that are, in part, defined by a sense of loss of control and compensatory behaviors to avoid weight gain. Impulsive behaviors are also common in individuals with BN, indicating more pervasive difficulties in behavioral self-regulation. Findings from functional and anatomical neuroimaging studies of individuals with BN suggest dysfunction in the dorsal frontostriatal circuits that support self-regulatory capacities and habit learning and in overlapping ventral circuits that support reward processing and reward-based learning. In this review, we describe the normal development of frontostriatal circuits and then present behavioral and neuroimaging data from adolescents and adults with BN. These data suggest that the abnormal maturation of frontostriatal circuits may contribute to the habitual binge-eating and purging behaviors of BN. Future longitudinal imaging studies will improve understanding of how these circuits contribute to the developmental trajectory of BN and will inform novel interventions that could target or prevent the impulsive and habit-like features of this disorder.

## Introduction

Bulimia Nervosa (BN) is characterized by the presence of binge-eating and compensatory behaviors, such as self-induced vomiting, which are intended to prevent weight gain (BN; American Psychiatric Association, 2000). BN affects 1–3% of women (American Psychiatric Association, 2000; Hudson et al., 2007), with higher lifetime prevalence rates of BN reported when updated (*DSM-5*) diagnostic criteria are used (Keski-Rahkonen et al., 2009). The disorder is associated with significant psychosocial impairment, medical complications, and high rates of comorbid psychopathology (Wonderlich and Mitchell, 1997; American Psychiatric Association, 2000). Although gold-standard, empirically supported psychotherapies exist for BN, these first-line treatments result in symptom abstinence in only 30–50% of treatment-completing patients (Mitchell et al., 2007; Wilson et al., 2007).

The identification of BN vulnerability and early maintenance factors is imperative. Numerous risk models for the development of BN exist [see Stice (2002) and Jacobi et al. (2004)], but these models have only recently begun to incorporate neurocognitive and imaging findings [e.g., Pearson et al. (2014)], and, to our knowledge, none comprehensively consider BN onset within the context of neurodevelopment. An improved understanding of the neural and behavioral bases of the disorder and its development may improve and inform treatment development and selection, while permitting the prediction of outcome. In this review, we survey behavioral and neuroimaging findings related to self-regulatory control and reward-based learning – two neurocognitive capacities that appear instrumental to the development and persistence of BN. We subsequently synthesize these findings in a working developmental pathophysiological model of BN, which highlights directions for future research.

### Self-regulatory control and reward-based learning

Self-regulatory control is required for many everyday actions to coordinate the decision to execute one behavior and inhibit another. Definitions of the concepts of self-regulatory control and “action inhibition” in the psychological literature continue to be refined. Inhibitory control or action inhibition may include only the process of inhibiting a pre-planned motor response, or may subsume a number of processes, including attention and interpretation of signals, decision-making, response selection, and action execution (Eagle et al., 2008). The concept of self-regulation is broader and encompasses these capacities, as well as the ability to regulate emotional responses and to inhibit temptations or impulses for immediate gratification in the service of waiting for larger, more delayed rewards (Mischel et al., 1989).

Whereas self-regulation encompasses the use of goals, hypotheses, and plans to direct behavior, reward-based learning relies solely on previous reinforcement history to direct behavior (Daw et al., 2005). We use the term “reward-based learning” to refer to both reward-processing functions, including the capacity to anticipate, respond to, and learn from reward outcomes, and habit (i.e., stimulus–response) learning, which involves the association of a behavior with antecedent, often contextual stimuli (Balleine and O’doherty, 2009). Reward-processing functions rely on ventral portions of frontostriatal circuits including orbitofrontal cortex (OFC), ventral striatum (VS), and connected mesolimbic areas (Haber and Knutson, 2009). Habit-learning specifically relies on the intact functioning of the dorsal striatum (Packard and Knowlton, 2002).

From a clinical perspective, individuals with BN demonstrate deficits in self-regulatory control. A sense of “loss of control (LOC)” is a defining feature of binge-eating episodes (Wolfe et al., 2009) and has, in fact, been suggested to be a more salient aspect of these episodes compared with the actual amount of food consumed during the episodes (Wolfe et al., 2009; Mond et al., 2010; Shomaker et al., 2010). These recurrent binge-eating episodes and the relatively high frequency of other co-occurring impulsive behaviors (Holderness et al., 1994; American Psychiatric Association, 2000; Paul et al., 2002; Kaltiala-Heino et al., 2003) suggest an impaired capacity for self-regulation in individuals with BN. As binge-eating behaviors often become repeated, maladaptive actions that follow a particular antecedent, such as negative affect (e.g., Haedt-Matt and Keel, 2011), abnormal reward-based, habit-learning processes may also be involved in BN pathophysiology.

### Frontostriatal circuits involved in self-regulation and reward-based learning

The administration of neurocognitive tasks in the scanning environment has permitted the characterization of frontostriatal circuits that connect prefrontal cortices with the striatum and support self-regulatory capacities and reward-based learning. These frontostriatal circuits lie within the broader cortico-striato-thalamo-cortical (CSTC) loops that relay information from cortical to subcortical and then back to cortical regions. At least five parallel CSTC loops have been identified, initiating from and projecting back to (1) the supplementary motor area (SMA), (2) the frontal eye fields (FEF), (3) the dorsolateral prefrontal cortex (DLPFC), (4) the lateral OFC, and (5) the anterior cingulate cortex (ACC; Alexander et al., 1986, 1990). The prefrontal cortical portions of these pathways have long been assigned an important role in the control of thoughts and behavior in accord with the pursuit of internal goals (Miller and Cohen, 2001). The first three of these loops, which involve the SMA, FEF, and DLPFC, pass through the dorsal striatum. The last two loops, which involve the lateral OFC and ACC, pass through the ventromedial striatum (Alexander et al., 1986, 1990).

Whereas dorsal frontostriatal circuits support self-regulatory functions and the dorsal striatum supports habit learning, more ventral circuits including OFC and VS are involved in reward-processing functions. Thus, while dorsal circuits may be especially relevant to the control over habitual behaviors that involve rewarding stimuli (such as binge eating), disturbances in ventral circuits may alter reward-processing functions that may also contribute to the binge-purge cycle.

## Development of Frontostriatal Circuits Underlying Self-Regulatory Capacities and Reward-Based Learning Functions in Healthy Individuals

Substantial evidence suggests that frontostriatal circuits support the capacity for self-regulation in both health (Marsh et al., 2006) and illness (Marsh et al., 2009c). In health, this capacity develops rapidly during childhood and adolescence, paralleling, and likely depending on, the maturation of frontostriatal circuits. Thus, disturbances in the maturation of these circuits and self-regulatory capacities likely contribute to the development of a variety of psychiatric disorders in which children and adolescents have difficulty regulating their thoughts, emotions, and behavior (Marsh et al., 2009c). In BN, functional deficits in frontostriatal circuits may leave urges (e.g., to continue eating or to engage in compensatory behaviors, such as self-induced vomiting) unchecked. The abnormal maturation of these circuits may underlie self-regulatory deficits in individuals with BN and, ultimately, the persistence of the disorder. Abnormal development of reward-based learning circuits could contribute to strong and early associations between binge-eating and purging behaviors and their antecedents, as well as an inability to learn from the negative consequences of past experiences and behaviors and/or an overvaluation of the reward associated with behaviors. In turn, individuals may repeat behaviors, such as binge eating or purging, until they crystallize into maladaptive habits. Therefore, the developmental trajectories of the striatal and mesolimbic regions that support reward-based learning and their interaction with frontostriatal control systems may be essential to the development of BN. As such, an updated understanding of the normal developmental trajectory of these circuits and capacities is crucial for the identification of BN risk factors and potential targets for early interventions.

### Normal development of frontostriatal control circuits

Functional magnetic resonance imaging (fMRI) data from healthy individuals indicate functional changes within frontostriatal circuits that underlie age-related improvements in the capacity for self-regulation (Casey et al., 1997; Rubia et al., 2000b, 2006; Luna et al., 2001; Adleman et al., 2002; Bunge et al., 2002; Tamm et al., 2002; Marsh et al., 2006). Increasing prefrontal activation from childhood to adulthood on tasks of self-regulatory control processes (Rubia et al., 2000a; Casey et al., 2002; Marsh et al., 2006) likely reflects age-related increases in synaptic pruning and myelination in the frontal lobe (Huttenlocher, 1979). Anatomical MRI studies have tracked changes in brain volumes, gray matter density, and cortical thickness longitudinally in healthy individuals (Giedd et al., 1999; Gogtay et al., 2004; Sowell et al., 2004). Findings suggest that prefrontal cortices, which mediate more advanced, higher-order functions such as self-regulation, mature later than areas supporting more basic cognitive functions, such as sensation and movement (Giedd et al., 1999; Gogtay et al., 2004; Sowell et al., 2004). In addition, diffusion tensor imaging (DTI) findings indicate that white matter tracts connecting PFC with cortical and subcortical regions continue to develop from adolescence to early adulthood (Asato et al., 2010), consistent with age-related increases in the strength of functional connections across these regions (Hwang et al., 2010). DTI findings also suggest that the anatomical development of frontostriatal circuits is associated with the development of inhibitory control. For example, in a sample of healthy individuals aged 7–31, increases in age were associated with decreased radial diffusivity along frontostriatal fibers, and decreased radial diffusivity was associated with enhanced and more efficient go/no-go task performance (Liston et al., 2006). Thus, age-related changes in the structure and connectivity of frontostriatal regions in healthy individuals likely contribute to the functional changes within frontostriatal circuits that underlie normal, developmental improvements in self-regulatory control.

### Normal development of reward-based learning functions

The dorsal striatum supports a “stimulus–response/habit” form of memory (Packard et al., 1989; Packard and White, 1991; Packard and Knowlton, 2002) that is anatomically and functionally distinct from the hippocampally mediated “cognitive/relational” learning and memory system (Eichenbaum, 2000). Tasks of probabilistic classification learning, which rely on the association of cues with behaviors, are commonly used to assess habit learning in human subjects. Rat, monkey, and human behavioral data suggest that striatally mediated habit-learning functions mature prior to hippocampally mediated memory [see Goodman et al. (2014) for comprehensive review]; however, the trajectory of normal striatal development in humans is less well understood compared with that of the hippocampus. Human hippocampal neurons continue to develop until they reach their adult-like appearance between 5 and 8 years of age (Eriksson et al., 1998), and hippocampal volumes continue to change through adolescence and adulthood (Østby et al., 2009). Anatomical data on the development of the dorsal striatum are somewhat mixed but suggest that volumes of the caudate and putamen peak prior to puberty and gradually decrease through adolescence and adulthood (Lenroot and Giedd, 2006).

The VS, which supports reward-processing functions, shares projections with the lateral OFC and the ACC (Haber and Knutson, 2009). Developmental imaging studies of reward processing have primarily assessed neural activity within specific regions of interest (i.e., ventrolateral and dorsolateral PFC, OFC, ACC, nucleus accumbens, and amygdala) in response to reward during probabilistic tasks of reward-related decision making. Findings from these studies are relatively inconsistent, with increasing age associated with increased activation of the ACC (Eshel et al., 2007); decreased (Ernst et al., 2005; Galvan et al., 2006; Cohen et al., 2010) or increased (Bjork et al., 2004; Van Leijenhorst et al., 2010) activation of the striatum; increased activation of the OFC (Galvan et al., 2006; Eshel et al., 2007), but not consistently (Van Leijenhorst et al., 2006; Cohen et al., 2010); and no change in dorsolateral PFC activation throughout development (Van Leijenhorst et al., 2006; Eshel et al., 2007). These inconsistent findings may be attributed to the inclusion of decision-making processes within paradigms designed to assess reward-processing functions, in turn influencing the specific brain regions that are recruited for performance (Manes et al., 2002).

Some fMRI data from healthy individuals suggest that the earlier functional maturation of reward-related compared with control-related brain circuitry contributes to sensation-seeking (i.e., risky) behaviors during adolescence (Ernst et al., 2006; Galvan et al., 2007; Casey et al., 2008). The development of inhibitory control tends to follow a linear pattern from childhood into late adolescence in healthy individuals [e.g., Williams et al. (1999)], consistent with fMRI findings of increasing activation of frontostriatal regions from childhood to adulthood on tasks of these processes (Rubia et al., 2000a, 2007; Casey et al., 2002; Marsh et al., 2006). In contrast, activation of VS and ventromedial PFC in response to rewards increases with increasing age, peaking during adolescence (between ages 13 and 17) but decreasing thereafter (Galvan et al., 2006; Van Leijenhorst et al., 2010). This mismatch in the developmental trajectories of reward and control-related circuitry may result in exaggerated emotional responses and vulnerability to highly rewarding behaviors and substances in adolescence (Casey and Jones, 2010). Indeed, the suppression of approach behaviors toward appetitive cues is more impaired in adolescents (age 13–17) compared with children and adults (Somerville et al., 2011). Behavioral findings of enhanced sensitivity to reinforcement (Tripp and Alsop, 1999) and greater value assignment to immediate compared with future rewards (Crone and Van Der Molen, 2004; Hooper et al., 2004) in adolescents compared with adults are consistent with the real-world general behavioral style of adolescents. Sensation-seeking behaviors (Martin et al., 2002) are prevalent in adolescents, further suggesting that the late maturation of the PFC may contribute to hypersensitivity to reward during adolescence. Further, the ability to learn from changes in the reward value of outcomes may be overwhelmed by better-developed stimulus–response learning in adolescence. This may set the stage for poor emotional and behavioral regulation and the development of rewarding, but maladaptive, habitual behaviors, such as the binge-eating and purging behaviors of BN.

The interaction between the development of dorsal frontostriatal control and ventral reward circuits may also, in part, explain why BN often emerges more commonly in females (Hudson et al., 2007). Longitudinal fMRI data suggest sex-specific patterns of developmental change in the functioning of prefrontal regions involved in inhibitory control functions (Ordaz et al., 2013). In one study, females aged 9–16, but not males, showed hyperactivation of prefrontal regions during response inhibition that decreased with increasing age, suggesting greater reliance on prefrontal cortices early in development in females. Those findings were interpreted to reflect a sex-specific compensatory approach during adolescence (Ordaz et al., 2013), when inhibitory control in response to appetitive cues is particularly impaired in both males and females (Somerville et al., 2011). If female relative to male reliance on PFC regions in early adolescence reflects compensation for inefficient processing within those regions, it may, in part, contribute to both the adolescent onset and female predominance of BN. Despite this possibility, imaging studies of BN have not yet included male participants, and additional data are needed to understand how sex differences in the developmental trajectory of frontostriatal circuits may explain why more females than males develop BN.

We suggest that understanding how the developmental trajectories of self-regulatory and reward-based learning functions, and the overlapping frontostriatal circuits that support these capacities, deviate from normal in BN may, in part, help elucidate the causes of the disorder. Furthermore, this knowledge may help determine how and when to intervene and thus prevent binge-eating and purging behaviors from crystallizing into maladaptive habits.

## Behavioral Findings of Self-Regulatory and Reward-Based Learning Deficits in BN

### Poor self-regulation in BN

Behavioral data on the self-regulatory capacities of individuals with BN are mixed. Here, we briefly review findings from studies that used a variety of tasks designed to measure different subcomponents of self-regulatory processes. Go/no-go tasks measure action restraint by requiring the execution (i.e., go) and inhibition (i.e., no-go) of a prepotent motor response (usually button pressing) via executive functions including decision-making, response selection, and response inhibition (Rubia et al., 2001). Somewhat similarly, antisaccade tasks require the suppression of the urge to saccade toward a suddenly appearing peripheral target (Hallett, 1978). In contrast, stop-signal tasks (SST) measure action cancellation by requiring responses to go stimuli unless an auditory or visual stop-signal appears (Logan et al., 1997; Eagle et al., 2008). The Simon Spatial Incompatibility task (Simon, 1969), which requires interference inhibition as opposed to response inhibition, and the Stroop task (Stroop, 1935), a classic test of color–word interference, are examples of commonly used tasks of conflict monitoring and resolution. The Stroop task requires individuals to override a prepotent response (word reading) to instead name the font color of printed words. The Simon task is a non-verbal analog of the Stroop that also requires ignoring a task-irrelevant feature of a stimulus (the side of the screen on which an arrow appears) when it conflicts with a more task-relevant one (the direction the arrow points). Tasks that measure the ability to resist temptations or deter impulses for immediate gratification have also been used to assess self-regulatory capacities. For example, the Iowa Gambling Task (IGT) requires foregoing immediate gains in the service of avoiding large future losses (Bechara et al., 1994). Of note, a recent meta-analysis (Wu et al., 2013) reported findings from 36 behavioral studies of inhibitory and interference control in individuals with eating disorders characterized by binge-eating behaviors. BN-specific impairments in general inhibitory control (i.e., data from the 24 studies that used neutral, non-food, or body-shape stimuli) were reported with a small effect size (*g* = −0.26). Greater impairments were observed with food or eating-related task stimuli (*g* = −0.67) and body or shape-related stimuli (*g* = −0.61). Of note, only 1 of the 439 individuals with BN included in this meta-analysis was male, precluding generalizability of these findings to males with BN. Here, we briefly review the findings from a selection of these studies.

Compared with healthy individuals, adults with BN responded impulsively with faster reaction times and more errors on a go/no-go task that included food and body-shape word stimuli cues as go and no-go stimuli (Mobbs et al., 2008). Similarly, adult women with BN responded more impulsively than healthy participants on a Simon Spatial Incompatibility task (Marsh et al., 2009a), and those with the most severe BN symptoms performed the worst. Findings from a meta-analysis of Stroop task data further suggest that individuals with BN have deficient control over attention and interference resolution (Dobson and Dozois, 2004). Other data suggest that compared to healthy participants, those with BN do not show improved IGT performance over time, suggesting that in addition to potential deficits in inhibiting responses, individuals with BN are less able (or prefer not) to delay reward (Boeka and Lokken, 2006; Liao et al., 2009). Performance on the IGT was also correlated with frequency of bulimic symptomatology such that those with more frequent eating disorder behaviors performed worst on the task (Boeka and Lokken, 2006). In contrast to these findings of self-regulatory impairments in BN, other studies report no deficits compared to healthy participants on go/no-go (Bruce et al., 2003; Rosval et al., 2006) and stop-signal (Claes et al., 2006) tasks.

Aside from differences in the samples studied, these differential findings across studies may be due to the use of very different paradigms to assess self-regulatory control processes that actually measure slightly different cognitive constructs. Although meta-analytic results indicate slight deficits in general inhibitory control and more pronounced deficits in the context of disorder-relevant stimuli (Wu et al., 2013), the tasks included in this analysis assessed different components of behavioral inhibition and self-regulation. Further, only one study has examined self-regulatory control in adolescents who met diagnostic criteria for BN (Marsh et al., 2011). The dearth of research in children and adolescents precludes understanding of how self-regulatory impairments develop and change over time in BN.

### Reward-based learning in BN

Two studies to date have investigated habit learning in BN using the Weather Prediction task, which requires the gradual learning of stimulus–response associations. Declarative memory of the previous trial is not as useful in improving performance compared with the information gleaned across many trials to predict rain or sunshine. Healthy subjects exhibit learning on this task, as their ability to predict the correct weather outcome gradually improves over blocks of trials (Knowlton et al., 1994). Findings from the two extant studies of habit learning in BN are inconsistent. One study reported comparable behavioral performance across adult (over age 18) BN and control groups (Celone et al., 2011). In contrast, results from 60 adults and adolescents with BN (aged 12–46) compared with 60 matched controls suggest impaired habit learning on the Weather task (Labouliere et al., 2014). This impairment, however, was more pronounced in the younger participants, among whom symptom severity was associated with poorer accuracy. Older participants responded faster (i.e., more impulsively) on the task, and symptom severity was associated with their impulsive response style (Labouliere et al., 2014).

Research investigating behavioral responses to non-food rewards in individuals with BN is in relatively nascent stages. A recent investigation of IGT performance in 63 participants with BN (all but one of whom was female) reported increased sensitivity to gains vs. losses (Chan et al., 2014). This altered sensitivity to rewards relative to punishment in BN may set the stage for repeated engagement in a myriad of highly rewarding behaviors, regardless of their negative consequences.

## Function and Structure of Frontostriatal Control Circuits in BN Across the Lifespan

Most existing functional neuroimaging studies of BN have investigated brain function at rest or under controlled conditions using body-shapes, eating, or food stimulus presentation to study symptom-related brain processes in adults with BN [e.g., Nozoe et al. (1995), Kaye et al. (2001), Frank et al. (2004), Uher et al. (2004), and Bohon and Stice (2011)]. Although the study of disorder-relevant stimuli is important, examinations of behavioral and neural underpinnings of more broadly defined cognitive functions permit understanding of BN pathology and development both in the context of normal development and in comparison with other psychiatric disorders. Findings from several recent studies of BN, which included both adolescent and adult participants, suggest impaired functioning of the frontostriatal circuits underlying self-regulatory control processes.

### Frontostriatal functioning in adolescents with BN

**Figure 1:**
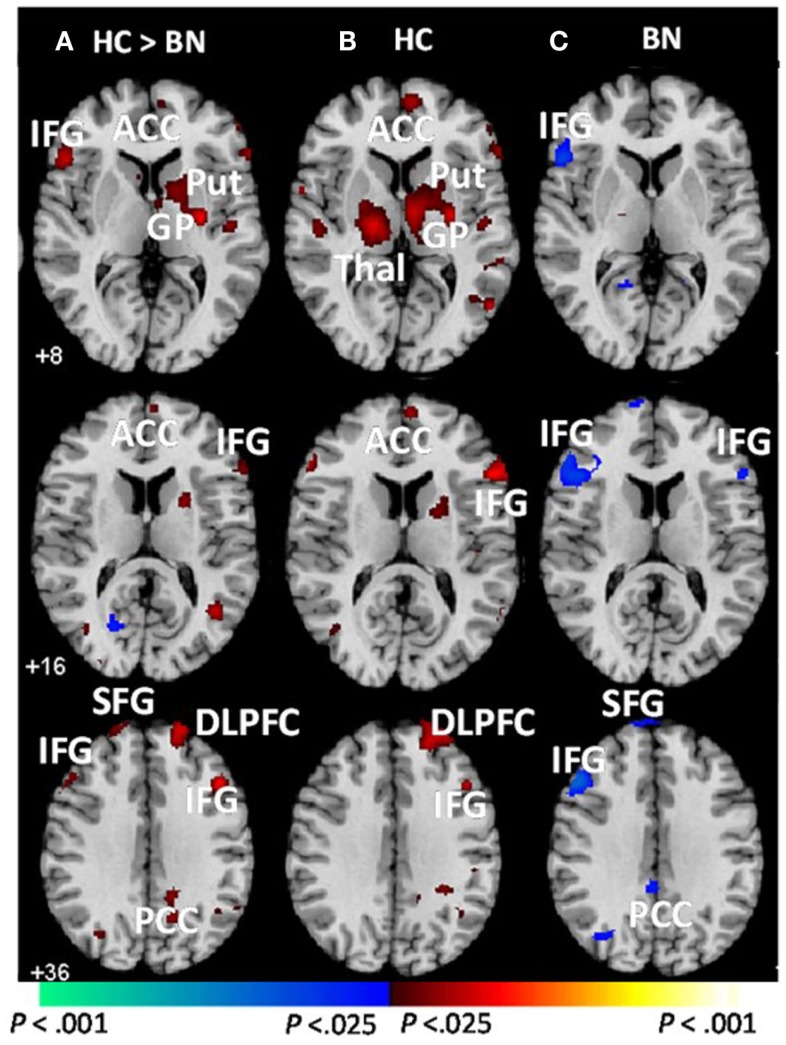
**Group average brain activations during correct trials [adapted from Marsh et al. (2011)]**. These are axial slices positioned inferiorly to superiorly (top to bottom). **(A)** Group-by-stimulus (congruent vs. incongruent) interactions were detected in frontostriatal regions (red). Main effects of stimulus condition (congruent vs. incongruent) are shown for the **(B)** healthy control and **(C)** BN adolescents. Increases in signal during correct incongruent relative to correct congruent trials are shown in red, and decreases are shown in blue. BN, bulimia nervosa; HC, healthy control; ACC, anterior cingulate cortex; DLPFC, dorsolateral prefrontal cortex; HI, hippocampus; IFG, inferior frontal gyrus; Put, putamen; GP, globus pallidus; Thal, thalamus; SFG, superior frontal gyrus; PCC, posterior cingulate cortex; MFG, medial frontal gyrus. Adapted and reprinted with permission from American Psychiatric Publishing.

Female adolescents with BN (aged 12–21; M age = 18.4, SD = 2.1) failed to activate frontostriatal circuits to the same extent as control participants when responding correctly to conflict stimuli on the Simon task (Marsh et al., 2011). In fact, girls with BN instead deactivated the left inferior frontal gyrus (IFG), as well as a neural system comprising the posterior cingulate cortex and the superior frontal gyrus [(Marsh et al., 2011); Figure 1]. These group differences in cortical and striatal regions were driven by the differential responses to stimuli preceded by conflict and non-conflict stimuli, respectively. The abnormal processing of the antecedent stimulus context in adolescents with BN may have conditioned their brain response to conflict differently than healthy adolescents, specifically in frontal regions. These findings indicate that functional disturbances in frontal portions of frontostriatal circuits arise early in the course of BN and may release eating and other behaviors from regulatory control. We are currently investigating Simon task-based connectivity between DLPFC and caudate nucleus in the healthy adolescents compared to those with BN. We predict that connectivity in dorsal frontostriatal systems may be abnormal in adolescents with BN; specifically, we hypothesize that deficient processing within frontal regions may contribute to deficient control of the DLPFC over the striatum, perhaps contributing to the habitual binge-eating behaviors that characterize BN.

Another study reported increased activation of right DLPFC, hypothalamus, bilateral precentral gyri, ACC, and middle and superior temporal gyri in a “binge/purge” group of adolescents (M age = 17.26, SD = 1.18) compared with controls during correct responses to no-go stimuli on a go/no-go task (Lock et al., 2011). Increased DLPFC activation may indicate compensation for inefficient PFC processing in the binge/purge group; however, these findings are in contrast with those of deficient prefrontal activation in adolescents with BN (Marsh et al., 2011). This inconsistency may be related to differences in the samples studied (the “binge/purge” group may not have met criteria for a diagnosis of BN, and 30% of this group were underweight), and the tasks used (go/no-go vs. Simon) that assess slightly difference capacities. Nevertheless, both sets of fMRI findings suggest aberrant PFC functioning associated with self-regulatory control in adolescents who binge eat and purge.

### Deficient frontostriatal functioning in adults with BN

**Figure 2:**
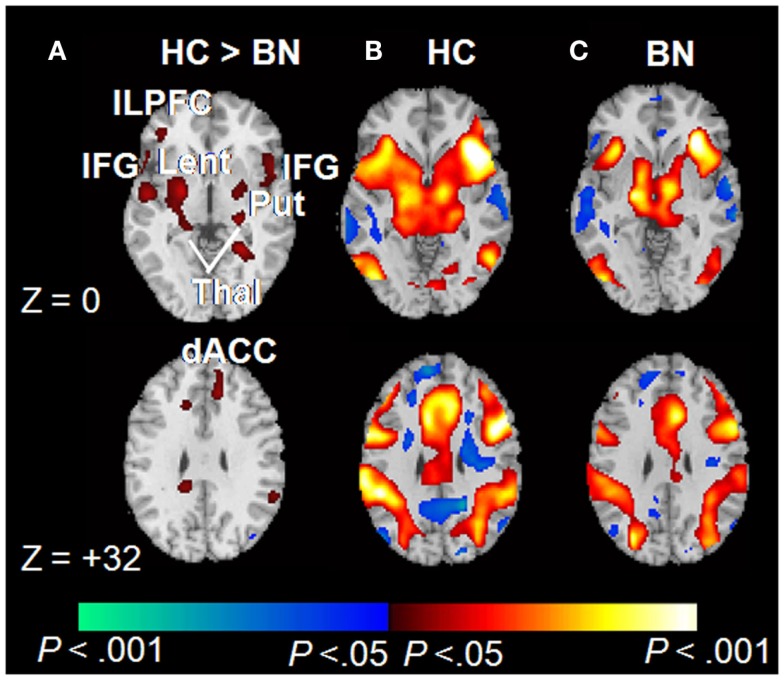
**Correct trials of the Simon spatial incompatibility task in healthy controls and individuals with BN [adapted from Marsh et al. (2009a)]**. **(A)** Group × stimulus (congruent vs. incongruent) interactions were observed in frontostriatal regions. **(B)** Main effects of congruent vs. incongruent stimuli within the healthy participants. Signal increases during incongruent relative to congruent trials are shown in red. **(C)** Main effects of stimulus condition among those with BN. dACC, dorsal anterior cingulate cortex; ILPFC, inferolateral prefrontal cortex; IFG, inferior frontal gyrus; Lent, lenticular nucleus; Put, putamen; Thal, thalamus. Copyright © (2009) American Medical Association. All rights reserved. Adapted and reprinted, with permission, from Marsh et al. (2009a). Personal use of this material is permitted. However, permission to reuse this material for any other purpose must be obtained from the American Medical Association.

Consistent with Simon task findings from adolescents with BN, adult women with BN (aged 19–46; M age = 25.7, SD = 7.0) also failed to activate frontostriatal circuits to the same degree as controls during correct responding to conflict stimuli (Figure 2). The number of objective bulimic episodes (i.e., large binge-eating episodes) in BN participants was inversely associated with frontostriatal activations, indicating reduced activity in the participants with the most severe symptoms. These findings suggest that self-regulatory processes are impaired in women with BN, and that this impairment may be directly related to their failure to engage frontostriatal circuits appropriately. Deficient functioning of these circuits may therefore contribute to impaired regulation of eating behaviors in both adults and adolescents.

### Structural abnormalities within frontostriatal circuits in BN

**Figure 3:**
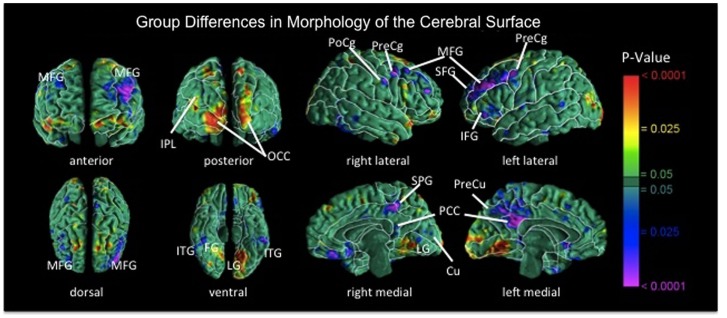
**Maps of group differences in morphological measures of the cerebral surface [reproduced from Marsh et al. (2013a)]**. The signed euclidean distances between points on the surfaces of the cortex for each participant and corresponding points on a template brain were compared statistically between the BN and control groups using linear regression at each voxel on the surface while covarying for age. BN group brains were significantly reduced bilaterally in medial frontal and precentral gyri, in superior and inferior frontal gyri of the left hemisphere, the postcentral gyrus of right hemisphere, and bilateral temporoparietal areas (*p*’s = 0.01–0.0001). MFG, medial frontal gyrus; PoCg, postcentral gyrus; PreCg, precentral gyrus; IFG, inferior frontal gyrus; SFG, superior frontal gyrus; IPL, inferior parietal lobule; OCC, occipital cortex; ITG, inferior temporal gyrus; FG, fusiform gyrus; SPG, superior parietal gyrus; PCC, posterior cingulate cortex; Cu, cuneus; PreCu, precuneus; LG, lingual gyrus.

In addition to functional deficits, recent findings suggest the presence of structural abnormalities within frontostriatal control circuits in BN. Surface based analyses revealed reductions in bilateral middle frontal and precentral gyri, right postcentral gyrus, and left lateral superior and inferior frontal gyri in individuals with BN compared with healthy participants [Figure 3; (Marsh et al., 2013a)]. These reductions in the BN group derived primarily from reductions of underlying white matter. In addition, reductions in inferior frontal regions correlated inversely with symptom severity, age (sample age ranged from 12 to 46), and Stroop interference scores in the BN group, suggesting that reductions specific to inferior frontal cortices may contribute to functional deficits in self-regulation and to the persistence of these deficits over development in BN. Findings from studies using voxel-based morphometry suggest reduced gray matter in bilateral caudate (Amianto et al., 2013; Frank et al., 2013) and dorsal putamen (Frank et al., 2013) in adults with BN compared with matched controls. Together, these anatomical MRI data indicate structural abnormalities within frontostriatal circuits in BN.

DTI data from adult women with and without BN (mean age of both groups was approximately 25) suggest reduced fractional anisotropy (FA) in bilateral corona radiata, a frontal white matter tract (Mettler et al., 2013). We are currently assessing group differences in age-related changes in white mater microstructure in adolescents and adult females with BN compared with matched controls. We hypothesize that the early disorganization of frontostriatal fiber tracts may, in part, contribute to the impaired functioning of these circuits and to the local volume reductions specific to frontal cortices over development in BN.

## Reward-Based Learning Functions in BN

While multimodal MRI data have begun to implicate impaired maturation of frontal cortex in the developmental trajectory of BN, the role of the striatum in BN development is less understood. fMRI findings from studies using symptom-relevant stimuli suggest that reward systems are dysfunctional in adults with BN. These studies are thoroughly reviewed elsewhere [e.g., Frank (2013) and Kaye et al. (2013)]. Unclear is whether activation of reward-related regions (especially VS) is increased (Radeloff et al., 2014) or decreased (Bohon and Stice, 2011; Frank, 2013) compared to healthy individuals in response to the anticipation or receipt of food rewards. Also unclear is whether these abnormalities are present in adolescents with BN.

Non-symptom-specific studies of striatally mediated functions in BN, including habit learning and reward-based memory or associative learning, are relatively few. One study reported increased activation of caudate and DLPFC in women with BN compared with controls during habit learning on the Weather Prediction Task despite comparable behavioral performances (Celone et al., 2011). These findings suggest that inefficient processing within frontostriatal circuits in individuals with BN could contribute to their overreliance on caudate and DLPFC in the service of habit learning. Recent preclinical data also indicate a potential role for the striatal-based habit-learning system in binge eating. Unlike control rats, rats with a history of binge-like consumption of palatable food failed to reduce their responding for a devalued food reward (Furlong et al., 2014), indicating a failure to update previous stimulus–response learning with new response-outcome information. The binge-eating group of rats whose habit learning overrode outcome-related information also demonstrated greater activity in the dorsolateral striatum [analogous to the human posterior lateral putamen (Balleine and O’doherty, 2009)], and their maladaptive behavior was corrected by chemically decreasing activation in this region (Furlong et al., 2014). Although more human research is necessary, these preclinical findings suggest that striatal alterations and reduced sensitivity to the devaluation of outcomes, particularly food-related outcomes, may contribute to the development and maintenance of habitual binge-eating behaviors in BN.

Beyond potential deficits in habit learning, altered reward processing and sensitivity may further complicate successful learning processes in BN and promote the development of habitual, maladaptive behaviors. For example, one study reported reduced activation in the insula, the ventral putamen, the amygdala, and the OFC of adult individuals with BN in response to unexpected omission and receipt of a conditioned sucrose solution stimulus (Frank et al., 2011). Binge-eating and purging frequency was inversely related to prediction errors, which suggests these behaviors may be related to aberrant frontostriatal responses to unexpected taste rewards.

**Figure 4:**
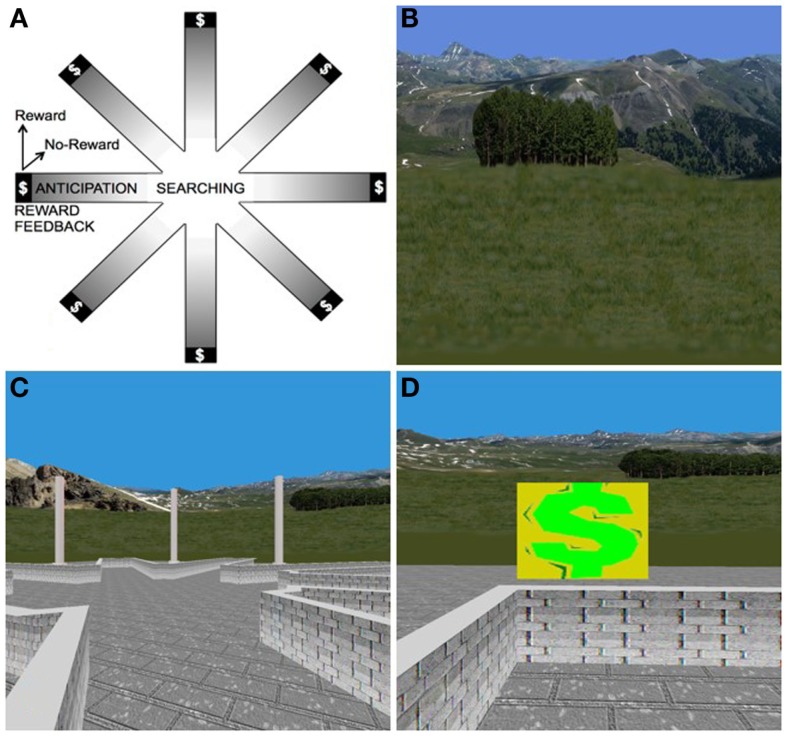
**The virtual-reality environment**. **(A)** Schematic of the virtual maze depicting the four events modeled: “searching,” “reward anticipation,” and the two types of reward feedback, “reward” and “no-reward.” **(B)** Some of the naturalistic spatial cues in the VR maze. **(C)** Participants’ view of the VR maze. **(D)** Baited area at the end of an arm, with $ indicating successful receipt of reward.

We recently used a novel, virtual-reality based paradigm to investigate the neural correlates of non-food, reward-based learning in adolescents with BN (Marsh et al., 2013b). This paradigm was developed to serve as a direct human analog of the radial arm maze experiments that defined the neural bases of stimulus–response and spatial learning systems in rodents (Packard et al., 1989; Packard and White, 1991). Thus, the paradigm consists of both spatial and stimulus–response learning tasks, each with a rigorously defined control condition (Marsh et al., 2010). Twenty-eight adolescents with BN (M ± SD age: 16.5 ± 2.2) and 26 age-matched healthy control participants (M ± SD age: 16.2 ± 2.1) used an MRI-compatible joystick to navigate and find hidden rewards inside an eight-arm radial maze with a central starting location and extra-maze cues visible from within (Figure 4). We compared groups in their patterns of brain activation associated with reward-processing during spatial learning vs. a control condition in which rewards were unexpected because they were allotted pseudo randomly and thus learning was experimentally prevented. Compared with healthy adolescents, individuals with BN failed to engage posterior hippocampus when searching the maze in the learning condition. In response to receiving unexpected rewards at the end of the maze arms, the adolescents with BN but not the healthy adolescents activated mesolimbic areas, including amygdala and anterior hippocampus. In addition, activation of the anterior hippocampus in response to unexpected rewards was greatest in individuals who had engaged in the greatest number of objective bulimic episodes in the 28 days prior to scanning. These preliminary findings may be interpreted in light of the differential roles of the posterior and anterior hippocampus in processing episodic memory and spatial information (posterior) and reward information (anterior), respectively (Behrendt, 2013).

Together, the extant findings suggest that portions of reward-based learning circuits in adolescents with BN may be hypersensitized to unexpected (non-food) rewards, whereas in adults, reward-based learning circuits are hypoactive in response to unexpected food rewards. Just as deficits in habit-learning appear to develop early in BN, prediction error signaling, and reward responses within ventral (anterior) midbrain areas may also develop early in adolescents with BN. This early development, in the context of slower PFC development, may place individuals at particular risk for the development of binge-eating behaviors. Repeated engagement in binge-eating behavior into adulthood may ultimately result in attenuated activation of reward-based learning circuits. Longitudinal studies of adolescents using paradigms that assess reward-based learning with food and non-food stimuli are needed to better understand how these processes and circuits contribute to the development and maintenance of BN.

## Developmental Pathophysiological Model of BN

Taken together, findings from neuroimaging studies suggest that the abnormal maturation of the overlapping frontostriatal and mesolimbic circuits supporting self-regulation and reward-based learning may contribute to the development and maintenance of BN. fMRI findings suggest that the failure of women with BN to engage frontostriatal systems appropriately contributes to their impairments in self-regulatory control (Marsh et al., 2009b), and that functional disturbances in frontostriatal regions arise early in adolescence (Lock et al., 2011; Marsh et al., 2011). Anatomical findings suggest that reductions on the surface of inferior frontal cortices may contribute to these functional deficits in self-regulation that persist over the course of this illness (Marsh et al., 2013a).

The later maturation of the frontal cortex compared with other brain regions in healthy development may be even more protracted in BN, thus contributing to these observed structural and functional abnormalities in individuals with the disorder. Similarly, we hypothesize that the earlier maturation of striatally mediated learning compared with hippocampally mediated learning may be more protracted in BN, thereby contributing to the development of entrenched binge-eating and purging behaviors. Frontostriatal disturbances may also affect reward-processing functions within mesocorticolimbic systems, thereby decreasing the rewarding relief that could normally result from eating and further driving urges to binge-eat (Bohon and Stice, 2011). Because learning requires a cross-talk between functional circuits, particularly reward and cognitive circuits (Haber, 2008), we suspect that these overlapping circuits are involved in the acquisition of the learned, habitual, binge-eating, and purging behaviors that characterize BN. Preclinical results suggest that over time, repeated engagement in binge eating may further alter neural circuitry that should update habits when rewards are devalued (Furlong et al., 2014), ultimately maintaining binge-eating behavior.

**Figure 5:**
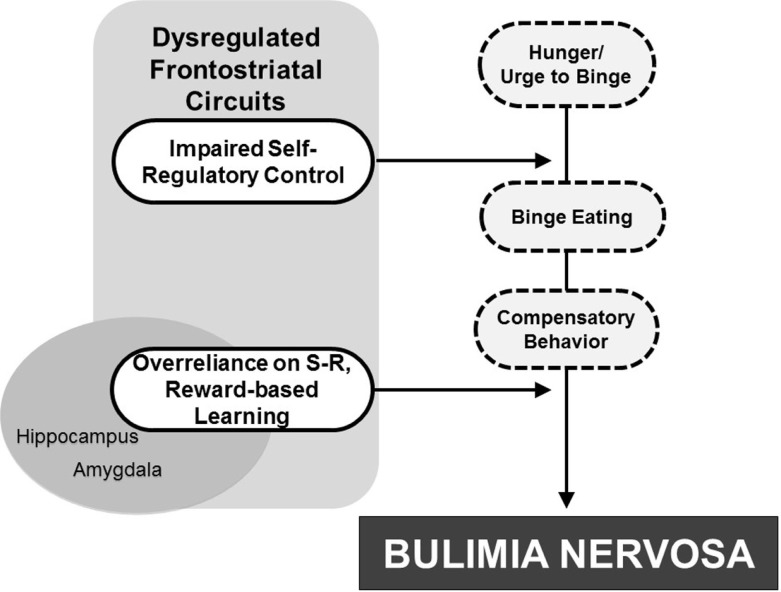
**Developmental pathophysiological model of BN**. The dysregulation of frontostriatal circuits likely contributes to an impaired capacity for self-regulatory control that interacts with hunger to release eating behavior from regulatory control. Attempts to compensate for weight gain contribute to purging behaviors. Interactions with reward-based learning systems, including striatal and mesolimbic regions, may then allow the binge-eating and purging behaviors to solidify into “habit-like” behaviors, ultimately contributing to BN development.

Concomitant with sociocultural determinants more thoroughly reviewed elsewhere and included in other models [e.g., Stice (2002) and Jacobi et al. (2004)], binge-eating behaviors may arise from or be facilitated by dysregulated frontostriatal control systems that release from control a preexisting vulnerability to the development of BN. This vulnerability may stem from altered functioning of serotonergic systems that produce both impulsivity and decreased satiety (Kaye, 2008). Urges to continue eating may be released inappropriately from control systems, thereby resulting in binge eating. Esthetic ideals of thinness and low distress tolerance [e.g., see Pearson et al. (2014) for review] may promote compensatory behaviors to counteract weight gain. Finally, interactions with abnormal reward-based learning systems may alter the processing of food rewards and allow binge-eating and purging behaviors to solidify as “habits,” thereby contributing to BN (Figure 5).

## Directions for Future Research

Relatively few neuroimaging studies have focused on BN, and even fewer studies have attempted to disentangle the neural and behavioral characteristics of BN that represent predisposing risk factors from those that are consequences of repeated eating disorder behaviors. Further, all of the BN participants included in the imaging studies reviewed here were female, precluding our understanding of frontostriatal structure or function among males with BN. Large, prospective, and longitudinal multimodal studies that include male and female individuals at the beginning of illness onset or adolescents at genetic and psychological risk for BN development are needed. Additional study of the dorsal striatal-based habit-learning system in adolescents may clarify how dysfunction in this system may contribute to the development of maladaptive binge-eating and compensatory behaviors. To date, neuroimaging studies of frontostriatal circuitry in BN have assessed the neural correlates of cognitive control or reward processing. No prior studies have assessed, within the same sample, the functioning of both systems, or how these systems may change with increasing age.

Further, more comprehensive analyses of the function, structure, and connectivity of overlapping frontostriatal circuits in adolescents and adults with BN are needed. For example, findings that reductions of inferior frontal cortices are associated with structural deficits on the Stroop task (Marsh et al., 2013a) suggest a structure–function relationship consistent with fMRI data (Uher et al., 2004; Marsh et al., 2009b, 2011; Lock et al., 2011; Mettler et al., 2013). Similarly, associations of striatal volume reductions with functional abnormalities on habit-learning and reward-processing tasks should be assessed in BN. Because the functioning of hippocampal-dependent processes is beginning to be studied in BN, structural analyses of this region and its subdivisions are also warranted. Thus, future research should include comprehensive, multimodal measures of the neural circuits that support self-regulatory control and reward-based learning. Future longitudinal imaging studies will enable us to identify potentially atypical neurodevelopmental trajectories within these circuits in BN and test our hypotheses regarding the protracted development of the frontal cortex and the earlier development of striatal vs. hippocampal learning systems. Although longitudinal studies will add to our understanding of the progression of BN, only studies of individuals at risk for the development of BN will allow understanding of the circuit-based disturbances that may cause the disorder.

To our knowledge, no studies have investigated circuit-based changes associated with specific therapeutic skills or treatment outcomes in BN. Nonetheless, the role of frontostriatal circuits in supporting self-regulatory capacities is well-understood, and some interventions have been found to impact the function of those circuits or portions of those circuits in individuals with anxiety disorders (Lueken et al., 2013) and borderline personality disorder (Goodman et al., 2014). Thus, the model we propose may offer some direction for future study of additions or alterations to evidence-based treatments for BN. A variety of existing cognitive-behavioral interventions for BN already target the improvement of self-regulatory strategies (e.g., Family Based Treatment, Enhanced Cognitive-Behavioral Therapy for BN, and Dialectical Behavior Therapy); however, potential adjunctive interventions may improve outcomes, especially if introduced early, at the onset of the disordered eating behavior. For example, data suggest that repeated and frequent rewards for correct responses on an antisaccade task improves the ability of healthy adolescents to recruit PFC during the engagement of inhibitory control (Geier et al., 2010). Thus, frequent rewards administered by therapists and caregivers, or personally administered, for abstinence from binge-eating and purging or for the use of alternative therapeutic skills, may contribute to more effective PFC recruitment – and hence, better self-regulatory control over disordered eating behaviors – in adolescents with BN. Other data suggest that neurocognitive “inhibitory control training” improves control in healthy individuals by enhancing preparatory prefrontal cortical and striatal activation (Berkman et al., 2014). Perhaps similar training could help patients with BN engage control over their eating behaviors. In addition, training focused on building associations of new stimulus–response pairs when outcomes are devalued might help patients decrease their reliance on maladaptive, habitual behaviors.

Pharmacological interventions such as Modafinil have been found to enhance PFC activation and improve cognitive control in both healthy adults and in psychiatric populations (e.g., individuals with schizophrenia; Minzenberg and Carter, 2007). Pairing such interventions with training or psychotherapy could also represent a promising combination of treatments for BN. Non-invasive brain stimulation, such as transcranial direct-current stimulation (tDCS) or repeated transcranial magnetic stimulation (rTMS), that could further enhance frontostriatal function, particularly prefrontal regulation of the striatum, may also hold promise as a treatment adjunct (Fregni et al., 2008; Walpoth et al., 2008; Van Den Eynde et al., 2010; Downar et al., 2013); however, the appropriate cortical target, intensity, frequency, and duration of stimulation is not yet known for most disorders [e.g., Feil et al. (2010)], and much less so for BN. Although far more imaging research is needed to fully understand the longitudinal course of frontostriatal structure and function among individuals at risk for developing BN, the results of such investigations could have significant implications for treatment approaches across the lifespan.

## Conflict of Interest Statement

The authors declare that the research was conducted in the absence of any commercial or financial relationships that could be construed as a potential conflict of interest.
